# Whole-Genome Analysis of Herbicide-Tolerant Mutant Rice Generated by *Agrobacterium*-Mediated Gene Targeting

**DOI:** 10.1093/pcp/pcu153

**Published:** 2014-11-04

**Authors:** Masaki Endo, Masahiko Kumagai, Ritsuko Motoyama, Harumi Sasaki-Yamagata, Satomi Mori-Hosokawa, Masao Hamada, Hiroyuki Kanamori, Yoshiaki Nagamura, Yuichi Katayose, Takeshi Itoh, Seiichi Toki

**Affiliations:** ^1^Agrogenomics Research Center, National Institute of Agrobiological Sciences, 2-1-2 Kannondai, Tsukuba, Ibaraki, 305-8602 Japan; ^2^Graduate School of Nanobioscience, Yokohama City University, 22-2 Seto, Kanazawa, Yokohama, 236-0027 Japan; ^3^Present address: Department of Biological Sciences, Graduate School of Science, University of Tokyo, 7-3-1 Hongo, Bunkyo-ku, Tokyo, 113-0033 Japan.

**Keywords:** Acetolactate synthase, Gene targeting, Herbicide tolerance, Rice

## Abstract

Gene targeting (GT) is a technique used to modify endogenous genes in target genomes precisely via homologous recombination (HR). Although GT plants are produced using genetic transformation techniques, if the difference between the endogenous and the modified gene is limited to point mutations, GT crops can be considered equivalent to non-genetically modified mutant crops generated by conventional mutagenesis techniques. However, it is difficult to guarantee the non-incorporation of DNA fragments from *Agrobacterium* in GT plants created by *Agrobacterium*-mediated GT despite screening with conventional Southern blot and/or PCR techniques. Here, we report a comprehensive analysis of herbicide-tolerant rice plants generated by inducing point mutations in the rice ALS gene via *Agrobacterium*-mediated GT. We performed genome comparative genomic hybridization (CGH) array analysis and whole-genome sequencing to evaluate the molecular composition of GT rice plants. Thus far, no integration of *Agrobacterium*-derived DNA fragments has been detected in GT rice plants. However, >1,000 single nucleotide polymorphisms (SNPs) and insertion/deletion (InDels) were found in GT plants. Among these mutations, 20–100 variants might have some effect on expression levels and/or protein function. Information about additive mutations should be useful in clearing out unwanted mutations by backcrossing.

## Introduction

Since the first report of gene targeting (GT) of an integrated antibiotic resistance gene in the tobacco genome (Paszkowski et al*.* 1988), various approaches aimed at homologous recombination (HR)-dependent GT have been attempted, and several instances of successful GT of an endogenous gene have been reported in crops (for a review, see Endo and Toki 2013). GT enables not only targeted knock-out but also precise modification of the gene of interest. If such modification provides a selectable phenotype to host plants, GT cells or GT plants can be selected without using an additional selection marker gene. Indeed, we have introduced targeted point mutations into the rice genome via GT; herbicide-tolerant rice (Endo et al. 2007) and tryptophan-hyperaccumulating rice (Saika et al. 2011) were generated successfully by inducing point mutations in the *acetolactate synthase* (ALS) gene and the gene encoding anthranilate synthase α-subunit 2 (ASA2), respectively. In both cases, truncated target genes containing desirable mutations were transformed to wild-type (WT) rice calli, and GT cells were identified by subsequent selection using an ALS-inhibiting herbicide and an analog of tryptophan. In these cases, random integration of GT vectors by non-homologous end-joining (NHEJ) often occurred (Endo et al. 2007).

PCR and Southern blot analysis are the standard methods most commonly used to detect integration of vector sequences. However, it is difficult to state categorically that no unintended integration of small exogenous DNA fragments has occurred in GT plants because an insertion of a few base pairs or <20 bp is below the detection limit of these techniques. Furthermore, it has been reported that *Agrobacterium* genomic DNA can also be transformed into the plant genome (Ülker et al. 2008, Kim and An 2012). Proving the existence or non-existence of DNA fragments derived from *Agrobacterium* is an important criterion in the context of regulation of GT plants. In this study, we investigated the molecular characterization of GT plants by array-based comparative genomic hybridization (CGH) and whole-genome sequencing in addition to PCR and Southern blot analysis. Genome CGH array is based on the use of differentially labeled test and reference genomic DNA samples that are hybridized simultaneously to DNA probes arrayed on a glass slide to allow high-resolution evaluation of differing DNA copy number between test and reference genomes. We used this method to detect sequences derived from the GT vector. On the other hand, whole-genome sequencing using next-generation sequencing (NGS) is another promising method for detecting the existence of sequences derived from *Agrobacterium* and somaclonal mutations. The results of our analysis indicate that the use of genome CGH array and whole-genome sequencing is useful in the evaluation of GT plants, and also demonstrate that our GT plants are comparable with mutant plants created by conventional non-GM breeding approaches.

## Results

### Characterization of GT plants by PCR and Southern blot analysis

**Fig. 1:**
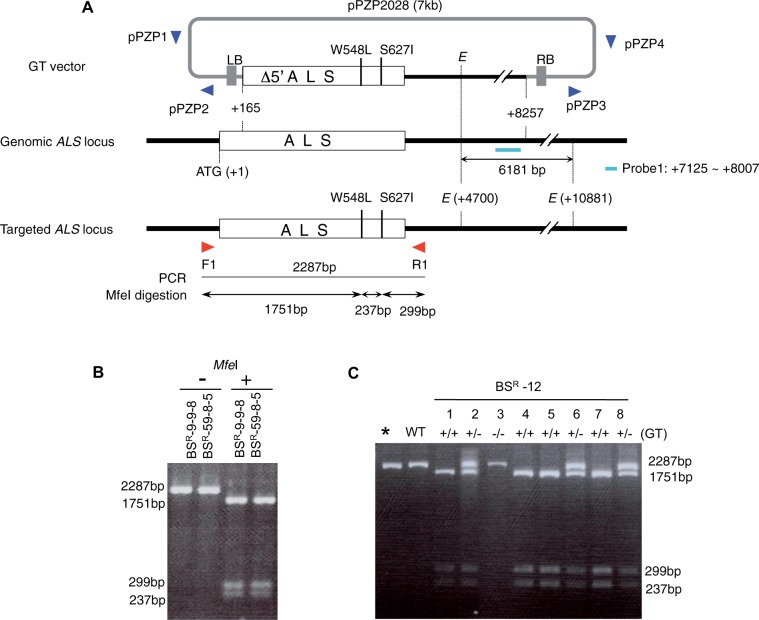
Strategy for T-DNA-mediated GT of the rice *ALS* locus and analysis of homologous recombination events in T_0_ plants. Schematic representation of GT events. The white boxes represent the coding region of the *ALS* gene. The thick black lines represent flanking rice genomic DNA. Left (LB) and right (RB) border sequences are represented by gray boxes. A sequence of 165 bp encoding 55 amino acids including the chloroplast-targeting signal is deleted in the T-DNA region of the GT vector, rendering the *ALS* gene non-functional. The two mutations (W548L and S627I) that confer BS tolerance exist on the GT vector. The W548L and S627I mutations create novel *Mfe*I restriction sites (CAATTG). The positions of primers (F1 and R1) used for PCR, and the expected size of PCR-amplified fragments and their *Mfe*I endonuclease digestion products are shown. *E*; EcoRI site. (B) PCR-*Mfe*I CAPS analysis of BS^R^-9-9-8 and BS^R^-59-8-5. PCR was performed using primers F1 and R1. The 2,287 bp PCR product resolves to three fragments (1,751, 299 and 237 bp) by *Mfe*I digestion when W548L and S627I mutations are induced in the endogenous ALS locus because W548L and S627I mutations create novel *Mfe*I sites. (C) PCR-*Mfe*I CAPS analysis of BS^R^-12-1 to BS^R^-12-8. *Non-digested PCR product of BS^R^-12-1; other lanes, *Mfe*I-digested PCR products.

In a previous study, we reported the successful production of novel herbicide-tolerant rice plants via introduction of two point mutations (W548L and S627I) into the rice *ALS* gene via HR-dependent GT using an *Agrobacterium* T-DNA-mediated transformation system (Endo et al*.* 2007). A schematic representation of this GT system is shown in Fig. 1A: the GT vector contains a truncated *ALS* coding sequence with two point mutations; tryptophan to leucine (TGG→TTG) at amino acid 548 (W548L), and serine to isoleucine (AGT→ATT) at amino acid 627 (S627I). Both mutations generate recognition sites for the restriction enzyme *Mfe*I (CAATTG). This GT vector was transferred into rice calli via *Agrobacterium*-mediated transformation as a template for HR. Because the chloroplast-targeting signal predicted by iPSORT (Bannai et al. 2002) in the N-terminal region of ALS is not present in the truncated *ALS* sequence on the T-DNA of the GT vector, random integration of T-DNA does not confer bispyribac (BS) tolerance, and functional and BS-tolerant ALS is created only by an HR event between the endogenous *ALS* locus and the T-DNA. From the 66 independent GT regenerated rice plants (T_0_) obtained in our previous study, we selected three independent GT lines (BS^R^-9, 12 and 59) for advanced sequence analysis in this study.

**Fig. 2:**
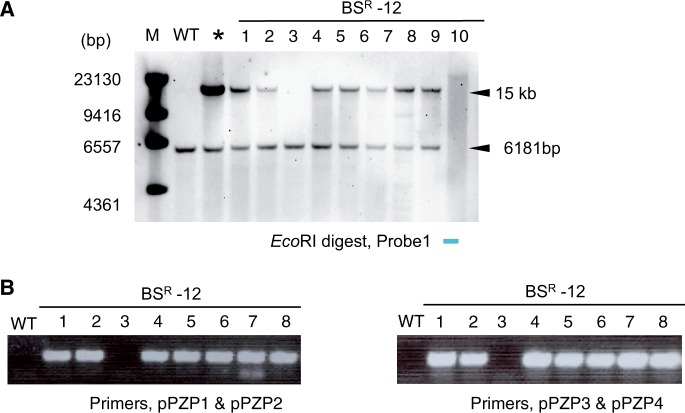
Detection of vector backbone sequence in siblings of BS^R^-12. (A) Southern blot analysis of T_2_ progeny of BS^R^-12 named BS^R^-12-1 to BS^R^-12-8. *GT vector was added to the WT. The position of the probe is shown in Fig. 1A. (B) PCR analysis using primers located near the LB and RB (Fig. 1A). Amplification of PCR products in BS^R^-12-1, 2 and 4–8 means that not only the T-DNA region but also the vector backbone sequence is integrated in these plants. The positions of the primers are shown in Fig. 1A.

**Fig. 3:**
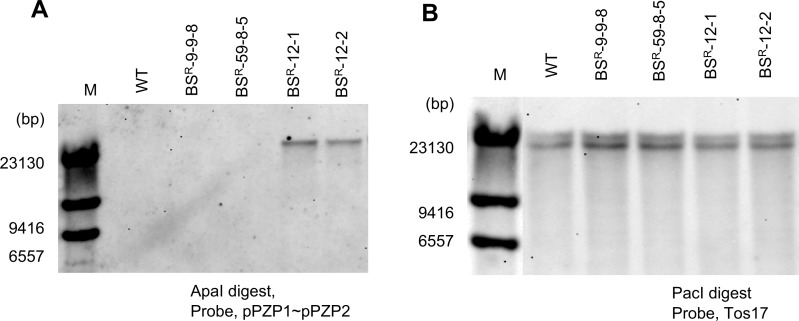
Confirmation of vector backbone insertion and Tos17 transposon copy number. (A) Southern blot analysis of *Apa*I-digested genomic DNA of BS^R^-9-9-8, BS^R^-59-8-5, BS^R^-12-1 and BS^R^-12-2 using a probe located on the vector backbone (Fig. 1A). (B) Southern blot analysis of BS^R^-9-9-8, BS^R^-59-8-5, BS^R^-12-1 and BS^R^-12-2 using a probe located on the Tos17 transposon.

In order to confirm the induction of point mutations in the endogenous *ALS* locus, we conducted cleaved amplified polymorphic sequence (CAPS) analysis using the restriction enzyme *Mfe*I. As shown in Fig. 1A, PCR amplification using primer F1, which anneals to the *ALS* promoter region (not present on the targeting construct), and primer R1, which anneals downstream of the *ALS* gene, yields a PCR product of 2,287 bp from both the WT and the GT *ALS* locus. Digestion of this PCR product with *Mfe*I distinguishes the WT from the GT *ALS* locus, since amplification products of the WT *ALS* locus cannot be digested by *Mfe*I but those of the GT *ALS* locus can be cleaved, yielding three fragments. From this analysis, we confirmed that modification of the *ALS* locus by GT was fixed to homozygote in the T_3_ generation of independent GT plants named BS^R^-9-9-8 and BS^R^-59-8-5 (Fig. 1B). In our previous study, we reported that no random integration of the GT vector occurred in progenitors of BS^R^-9-9-8 and BS^R^-59-8-5 (BS^R^-9 and BS^R^-59 in Endo et al. 2007). In the case of BS^R^-12, we found segregation of WT and mutated *ALS* in T_2_ plants (Fig. 1C). Southern blot analysis of T_2_ progeny of BS^R^-12 using a probe located on the rice genome sequence existing in the GT vector (Fig. 1A) gave a signal of 6,181 bp corresponding to the endogenous locus existing in all BS^R^-12 progeny, and a segregated signal around 15 kb (Fig. 2A). Taken together, the results of PCR-*Mfe*I CAPS analysis (Fig. 1C) revealed not only induction of W548L and S627I mutations in the endogenous *ALS* locus by HR-mediated GT but also random integration of the GT vector by NHEJ, which seemed to have occurred simultaneously in BS^R^-12. PCR analysis using primer sets that anneal to the vector backbone pPZP2028 revealed that vector backbone sequence, at least near the left (LB) and right (RB) border, was incorporated into BS^R^-12 (Fig. 2B). To reconfirm the presence or absence of vector backbone sequence in BS^R^-9-9-8, BS^R^-59-8-5, BS^R^-12-1 and BS^R^-12-2, we conducted Southern blot analysis using a probe located on the outside of the T-DNA border sequence (Fig. 1A, pPZP1-pPZP2). WT, BS^R^-9-9-8 and BS^R^-59-8-5 did not show any signal, but BS^R^-12-1 and BS^R^-12-2 showed a single band (Fig. 3A).

Transposition of transposable elements is one of the causes of genome instability. Among 32 transposon families in rice, Tos17 is known as the most active transposable element. Because the activity of retrotransposon Tos17 is associated with tissue culture (Hirochika et al. 1996), we assessed the copy number of Tos17 in our GT rice plants by Southern blot analysis. The results of this analysis revealed that the Tos17 copy number (two in Nipponbare) remained unchanged in GT plants (Fig. 3B).

### Revealing integration of GT vector sequences by genome CGH array

Results of PCR and Southern blot analyses (Fig. 3A) suggested that random integration of the GT vector had not occurred in BS^R^-9-9-8 and BS^R^-59-8-5. However, we are unsure how small an insertion can be detected by PCR and Southern blot analysis. So we conducted genome CGH array analysis using a custom-made tiling array in which the entire GT vector sequences were covered by overlapping 60 bp probes (Supplementary Fig. S1).

Array No. 1, WT (Cy5) vs. WT (Cy3), is a negative control experiment to show that the different fluorophores did not affect signal intensity. Array No. 2 is a positive control experiment using a Cy5-labeled DNA sample, in which the GT vector was mixed with WT rice genomic DNA at a ratio of one copy of GT vector to one copy of rice genome. Arrays Nos. 3 and 4 are additional positive control experiments using Cy5-labeled DNAs of 12-1-1 (No. 3) and 12-1-2 (No. 4), which are siblings of the same plant but containing the GT vector sequence in their genome. Arrays Nos. 5 and 6 represent the actual experiment, using BS^R^-9-9-8 and BS^R^-59-8-5, respectively. A scatter plot of these CGH array analyses is shown in Supplementary Fig. S2.

**Fig. 4:**
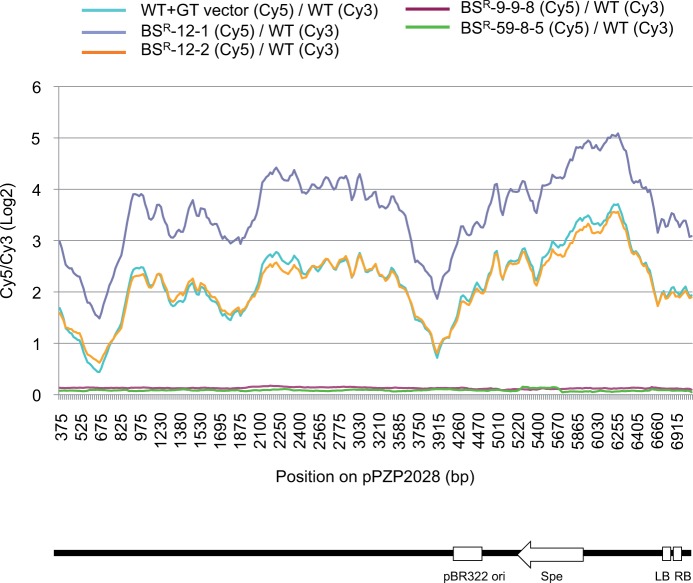
Array CGH ratio plots for the forward strand of binary vector pPZP2028. The log_2_ ratios of Cy5/Cy3 using a moving average of 20 adjacent probes (1.2 kb) were plotted against the cumulative kilobase pairs position. Locations of pBR322 ori, Spectinomycin resistance gene, LB and RB sequence existing on pPZP2028 are shown below the graph.

To facilitate visualization of genome CGH array data according to probe derivation, we mapped the Cy5/Cy3 ratio of probes according to their position on pPZP2028 (Fig. 4; Supplementary Fig. S3) and rice genomic DNA (Supplementary Fig. S4). In the case of pPZP2028, BS^R^-12-2 and a mixture of the GT vector and WT genomic DNA yielded approximately similar traces, and BS^R^-12-1 showed a similar shape to BS^R^-12-2 but the Cy5/Cy3 ratio was doubled (Fig. 4). These results mean that entire vector backbone sequences were integrated in BS^R^-12, and that these became fixed as homozygote in BS^R^-12-1 and existed as a heterozygote state in BS^R^-12-2. Despite the fact that the amount of DNA corresponding to each probe on pPZP2028 is the same in the mixture of GT vector and WT genomic DNA, the Cy5/Cy3 ratio differed among probes. This may be due to non-specific hybridization with rice genomic DNA, different binding magnitudes due to different GC content of probes, or biases. The use of increasing moving averages is effective for smoothing out consecutive data because background signal variation from one probe to another is normalized by adjacent probes (moving average 1, 5, 10 probe equivalent to 60, 300, 600 bp, Supplementary Fig. S3; moving average 20 probes equivalent to 1.2 kb, Fig. 4). However, excessive enlargement of the moving average increases the risk of overlooking small insertions. In the case of probes on the rice genomic sequence, the Cy5/Cy3 ratio in probes corresponding to the region located on the GT vector was significantly higher in the WT rice genome mixed with GT vector (WT + GT vector), BS^R^-12-1 and BS^R^-12-2 (Supplementary Fig. S4). In the case of BS^R^-9-9-8 and BS^R^-59-8-5, the log_2_ ratio of Cy5/Cy3 was around 0 for all probes. In all events, the Cy5/Cy3 ratios of probes located on the GT vector in BS^R^-9-9-8 and BS^R^-59-8-5 were markedly lower than those of positive controls (WT + GT vector, BS^R^-12-1 and BS^R^-12-2). We concluded that pPZP2028 sequences were not integrated in BS^R^-9-9-8 and BS^R^-59-8-5.

### Detection of sequences derived from *Agrobacterium* by whole-genome sequencing

Next, we conducted whole-genome sequencing to reveal sequences derived from *Agrobacterium* and to evaluate somaclonal mutations. Using a next-generation sequencer, the Illumina GAIIx, 48,091,545, 26,872,357, 56,205,201 and 43,374,403 crude sequencing runs of short reads (average 72 bp) were obtained covering BS^R^-12-1, BS^R^-12-2, BS^R^-9-9-8 and BS^R^-59-8-5, respectively. After trimming of low quality bases at both the 5′ and 3′ ends of crude sequences, and discarding reads that were too short (<20 bp), 47,857,496 (BS^R^-12-1), 26,777,904 (BS^R^-12-2), 55,633,580 (BS^R^-9-9-8) and 43,011,428 (BS^R^-59-8-5) reads, respectively, remained. Sequencing at a coverage equivalent to 9.3, 5.2, 10.8 and 8.3 times the size of the rice genome was conducted for BS^R^-12-1, BS^R^-12-2, BS^R^-9-9-8 and BS^R^-5-8-5, respectively. Using these sequence data, a BLAST search of sequences existing in *Agrobacterium* was performed. C58 genomic DNA (linear, accession No. NC_003063.2; circular, accession No. NC_00306j2.2), C58 plasmid At (accession No. NC_003064.2), Ti plasmid (accession No. NC_010929.1) and binary vector pPZP2028 [a derivative of pPZP202 (Hajdukiewicz et al. 1994). accession No. U10461.1), with the addition of rare restriction (*Asc*I/*Pac*I) sites to both ends of the multicloning site of pPZP202 sequences, were used as references. Sequences of binary vector pPZP2028 were detected in BS^R^-12-1 and BS^R^-12-2 but not in BS^R^-9-9-8 and BS^R^-59-8-5. No other sequences derived from *Agrobacterium* were found in any sequenced samples (Supplementary Table S1).

### Genetic variation in WT Nipponbare used for GT

To evaluate single nucleotide polymorphisms (SNPs) and short insertions/deletions (InDels), short reads were mapped onto the Nipponbare genome (IRGSP-1.0) using Burrows–Wheeler alignment tool (BWA) software (Li and Durbin 2009). The distribution of the read depth is shown in Supplementary Fig. S5. The peaks of sequencing depth of BS^R^-12-1, BS^R^-12-2, BS^R^-9-9-8 and BS^R^-59-8-5 were 8, 4, 6 and 10, respectively. Over 12,000 variants were called in every four samples (Supplementary Table S2). Variants commonly detected in more than two samples, except the combination of BS^R^-12-1 and BS^R^-12-2, might be due to polymorphism between the Nipponbare used for making a reference genome sequence and the Nipponbare used for generating GT plants. In this context, 10,757 SNPs and 1,556 InDels seemed to exist as polymorphisms in our Nipponbare line (Supplementary Table S2).

### Detection of line-specific SNPs and InDels

After removing these common variants, sample-specific variants were validated. When variants detected by two or more reads with quality call ≥10 (SAMtools) were selected, 2,556, 1,441, 1,488 and 989 line-specific variants were found in BS^R^-12-1, BS^R^-12-2, BS^R^-9-9-8 and BS^R^-59-8-5, respectively (Supplementary Table S3). As the quality score at SAMtools increased, the evaluated numbers of variants, especially hetero SNPs and hetero InDels, decreased. To improve the reliability of variations, line-specific variants existing in the region called by all four samples were selected. When variants called by two or more reads with quality score 10 at SAMtools (DP2Q10) were selected, 1,336, 855, 826 and 630 variants were called in BS^R^-12-1, BS^R^-12-2, BS^R^-9-9-8 and BS^R^-59-8-5, respectively, (Table 1, column total raw). Meanwhile, 288, 254, 245 and 183 total variants with quality score 20 at SAMtools (DP4Q20) were called in each sample by four or more reads. Strict search criteria reduced the call of false positives but reduced coverage of the genome. In fact, the average chromosome coverage shared by the four sequenced samples at DP2Q10 was 70.8%, and that at DP4Q20 was 46% (Supplementary Table S4). To validate the reliability of NGS, we reconfirmed SNPs detected in BS^R^-9-9-8 by dye terminator sequencing. We aligned SNPs in order of quality score, which indicated the reliability of variants at SAMtools. Among 45 homozygous SNP candidates, 12 homozygous SNP candidates with low quality score were sequenced. As a result, six SNPs from the bottom were not detected and six from the top were detected. So we estimated that 39 from the top quality score out of 45 homozygous SNP candidates might actually exist. Among 121 heterozygous SNP candidates, 48 heterozygous SNP candidates with high quality score were sequenced. As a result, 35 SNPs from the bottom were not detected and 13 from the top were detected. So we estimated that 13 from the top quality score out of 121 heterozygous SNPs candidates actually exist. One-quarter of heterozygous mutations fixed to homozygote in the next generation by the process of self-fertilization, theoretically. Therefore, 43.75% and 12.5% of the original mutations in T_0_ plants should have been homozygous and heterozygous in the T_3_ generation plant BS^R^-9-9-8, respectively. Because the ratio of homozygous SNPs (39) to heterozygous SNPs (13) estimated by dye terminator methods was similar to the expected ratio and the majority of homo SNPs called by NGS were detected by dye terminator methods, we concluded that the number of homozygous SNPs called by NGS was reliable.

**Table 1:** Number of SNPs and InDels*^a^*

Sample (generation)	Depth:quality*^b^*	Homo SNPs		Hetero SNPs		Homo InDels		Hetero InDels		Total		T0*^d^*			
		Raw	Corrected*^c^*	Raw	Corrected	Raw	Corrected	Raw	Corrected	Raw	Corrected	SNPs	InDels	Total	Average
BS^R^-12-1 (T2)	DP2Q10	59	84	894	1,277	63	90	320	457	1,336	1,887	225	240	465	501
BS^R^-12-2 (T2)	DP2Q10	88	126	575	821	53	76	169	241	885	1250	335	202	537	501
BS^R^-9-9-8 (T3)	DP2Q10	100	143	487	696	43	61	196	280	826	1167	327	140	467	
BS^R^-59-8-5 (T3)	DP2Q10	117	167	352	503	29	41	132	189	630	890	382	95	477	
BS^R^-12-1 (T2)	DP4Q20	34	74	156	339	17	37	81	176	288	626	197	99	296	307
BS^R^-12-2 (T2)	DP4Q20	37	80	152	330	18	39	47	102	254	552	214	104	319	307
BS^R^-9-9-8 (T3)	DP4Q20	45	98	121	263	15	33	64	139	245	533	224	75	298	
BS^R^-59-8-5 (T3)	DP4Q20	49	107	90	196	13	28	31	67	183	398	243	65	308	

*^a^* Number of sample-specific variants detected in the region called by all four samples.

*^b^* Validated depth and quality. DP2Q10: reads filled by depth ≥2, quality ≥10 in SAMtools. DP4Q20: reads filled by depth ≥4, quality ≥20 in SAMtools

*^c^* Number of variants, corrected by coverage of the genome (Supplementary Table S4).

*^d^* Expected number of SNPs and InDels in T_0_ calli calculated from the corrected number of homo SNPs, homo InDels and generation.

### Number of mutations induced during creation of GT plants

From the number of homozygous variants in NGS, genome coverage and the generation of analyzed plants, the expected number of mutations induced by the process of creating GT plants was estimated. As a result, 465, 537, 467 and 477 variants (DP2Q10) or 296, 319, 298 and 308 variants (DP4Q20) were called as expected mutations existing in T_0_ plant of BS^R^-12-1, BS^R^-12-2, BS^R^-9-9-8 and BS^R^-59-8-5, respectively. Because BS^R^-12-1 and BS^R^-12-2 are siblings of the same T_0_ plant, averages of the number of expected mutations in these two samples, 501 (DP2Q10) and 307 (DP4Q20), were adopted as expected mutations existing in BS^R^-12. The estimated number of mutations induced during cell culture and transformation processes in all three GT lines converged at around 500 (DP2Q10) and 300 (DP4Q20) (Table 1, column ‘T_0_ total’). Since the genome size of rice is 3.9×10^8^ bp, the somaclonal mutation rate in these plants is estimated to be around 1×10^–6^ bases per rice genome. Miyao et al. (2012) reported a somaclonal mutation rate of 1.7×10^–6^ in rice after 5 months of cell culture. Because the number of somaclonal mutations is affected by the period of cell culture, observing fewer somaclonal mutations in our GT plants undergoing 2 months of cell culture seems reasonable.

### Characterization of SNPs

All types of base changes were detected (Table 2), with the most frequent being from C to T. Changes from C to A and A to G were also observed with high frequency. The molecular spectrum of somaclonal variation detected in our study is analogous to the spectrum of spontaneous mutation in Arabidopsis, except that the C to T change is more prominent in Arabidopsis (Ossowski et al. 2010). The average ratio of transition to transversions in the four analyzed plants was 1.115, which is not so very different from the ratio (1.1) seen in regenerated rice plants without transformation (Miyao et al. 2012) but differs from the ratio (2.41) seen in sexually propagated Arabidopsis plants (Ossowski et al. 2010). Prediction of the potential SNPs and InDels indicated that 60, 25, 19 and 22 mutations in BS^R^-12-1, BS^R^-12-2, BS^R^-9-9-8 and BS^R^-59-8-5 may affect the quantity and/or quality of proteins (Table 3).

**Table 2:** Pattern of homozygous SNPs

SNP pattern	BS^R^-12-1 (T2)		BS^R^-12-2 (T2)		BS^R^-9-9-8 (T3)		BS^R^-59-8-5 (T3)	
	No.*^a^*	%	No.*^a^*	%	No.*^a^*	%	No.*^a^*	%
Homozygous transition								
AT→GC	6	10.2	10	11.4	22	22	18	15.4
GC→AT	24	40.7	36	40.9	37	37	37	31.6
Homozygous transversion								
AT→CG	2	3.4	8	9.1	4	4	5	4.3
AT→TA	9	15.3	12	13.6	11	11	10	8.5
GC→CG	6	10.2	8	9.1	8	8	7	6.0
GC→TA	12	20.3	14	15.9	18	18	40	34.2
Total	59		88		100		117	
Ti	30	50.8	46	52.3	59	59	55	47.0
Tv	29	49.2	42	47.7	41	41	62	53.0
Ti/Tv	1.03		1.1		1.44		0.89	

*^a^* SNPs called by two or more reads with quality score 10 at SAMtools (DP2Q10).

**Table 3:** Annotation of variants by snpEff

Effect of SNPs and InDel	BS^R^-12-1 (T2)		BS^R^-12-2 (T2)		BS^R^-9-9-8 (T3)		BS^R^-59-8-5 (T3)	
	Count	Percentage	Count	Percentage	Count	Percentage	Count	Percentage
FRAME_SHIFT	**6**	0.21%	**2**	0.11%	**0**	0%	**0**	0%
NON_SYNONYMOUS_CODING	**37**	1.31%	**17**	0.90%	**14**	0.84%	**20**	1.47%
CODON_CHANGE_PLUS_CODON_DELETION	**0**	0%	**1**	0.05%	**0**	0%	**0**	0%
START_GAINED	**3**	0.11%	**3**	0.16%	**2**	0.12%	**1**	0.07%
START_LOST	**0**	0%	**0**	0%	**2**	0.12%	**0**	0%
STOP_GAINED	**12**	0.43%	**1**	0.05%	**0**	0%	**1**	0.07%
SPLICE_SITE_ACCEPTOR	**1**	0.04%	**1**	0.05%	**1**	0.06%	**0**	0%
SPLICE_SITE_DONOR	**1**	0.04%	**0**	0%	**0**	0%	**0**	0%
SYNONYMOUS_CODING	13	0.46%	7	0.37%	5	0.30%	3	0.22%
EXON	11	0.39%	2	0.11%	5	0.30%	2	0.15%
INTRON	166	5.89%	136	7.20%	99	5.95%	76	5.57%
UTR_3_PRIME	40	1.42%	32	1.70%	22	1.32%	14	1.03%
UTR_5_PRIME	21	0.75%	24	1.27%	11	0.66%	20	1.47%
UPSTREAM	722	25.60%	477	25.27%	395	23.72%	365	26.76%
DOWNSTREAM	690	24.47%	474	25.11%	414	24.87%	341	25%
INTERGENIC	1,097	38.90%	711	37.66%	695	41.74%	521	38.20%
Total	2820		1888		1665		1364	

Variants called by two or more reads with quality score 10 at SAMtools (DP2Q10) were annotated.

Mutations predicted to affect the quantity and/or quality of proteins are shown in bold.

## Discussion

Modification of an endogenous gene without integration of any exogenous gene sequence is one of the main advantages of GT. From this point of view, confirmation of the presence or absence of sequences derived from *Agrobacterium* is a key issue in the evaluation of GT plants.

Southern blot analysis, PCR, genome CGH array analysis and whole-genome sequencing are based on different principles of detecting sequences of interest. Southern blot and PCR are useful as a first screening to eliminate GT lines in which random integration of the GT vector has occurred. In fact, Southern blot analysis using a probe located on the T-DNA region (Endo et al. 2007) and vector backbone (Fig. 3A) revealed that random integration of the GT vector occurred in BS^R^-12-1 and BS^R^-12-2 but not in BS^R^-9-9-8 and BS^R^-59-8-5. However, details of insertion, such as copy number of integrated DNA, and the precise location of the integrated region cannot be clarified by Southern blot analysis.

In genome CGH array, DNAs of test and reference plants are labeled by different fluorescent dyes and hybridized directly to an oligo-array in which the sequences of interest are spotted as a mass of short oligonucleotide probes. In our case, the whole vector backbone sequence and the rice genome including 8 kb existing on the T-DNA region of the GT vector were covered by multiple overlapping probes, hence minimizing the risk of overlooking an insertion. Upon hybridization with a combination of WT and BS^R^-12-1 or a combination of WT and BS^R^-12-2, BS^R^-12-1 or BS^R^-12-2 specific signals were detected with probes corresponding to the whole GT vector sequence, and the signal intensity of BS^R^-12-1 was exactly double that of BS^R^-12-2 across all GT vector sequences (Fig. 4; Supplementary Figs. S2–S4). Thus, genome CGH array analysis seems to be useful not only for identifying inserted sequences but also for distinguishing copy number variations. On the other hand, even if the amount of complemented DNA is the same, signal intensity can vary depending on the probe used. In the case of expression arrays, probes can be selected carefully so that they all hybridize with similar efficiencies at a given temperature, and cross-hybridization with other known parts of the transcriptome are minimized. In addition, gene expression probe sets are selected to provide a linear signal response with respect to target concentration. On the other hand, when tiling the entire GT vector sequence mechanically as probes for genome CGH array, sequence specificity and the melting temperature (T_m_) of probes cannot be equalized. Thus, probe signal intensity differs widely even with the same amount of compatible DNA due to different binding strength and/or non-specific hybridization. Averaging of signals of overlapping multiprobes is effective for alleviating variation due to individual probe sequences (Supplementary Fig. S3) but, conversely, makes it difficult to detect small insertions. Nonetheless, the log_2_ ratio of BS^R^-9-9-8/WT and BS^R^-59-8-5/WT does not overlap with that of BS^R^-12-2/WT even using a moving average of 60 bp corresponding to a single probe. Because running genome CGH array is cheaper than whole-genome sequencing, array-based detection of vector sequences is still a valuable method for screening GT lines of interest.

Whole-genome sequencing offers the means to study genomic differences more exhaustively, i.e. not only the existence of exogenous sequences but also the occurrence of unpredicted modifications such as somaclonal mutations. We performed an average of 7× coverage whole-genome sequencing using GT plants. Homology searches with sequences derived from *Agrobacterium* confirmed that these sequences did not exist in BS^R^-9-9-8 and BS^R^-59-8-5 (Supplementary Table S1). On the other hand, >300 somaclonal mutations were predicted to be induced during GT plant generation (Table 1). Among detected mutations, 60, 25, 19 and 22 mutations were predicted to affect the quantity and/or quality of proteins in BS^R^-12-1, BS^R^-12-2, BS^R^-9-9-8 and BS^R^-59-8-5, respectively (Table 3, shown in bold). The existence of somaclonal mutation is not relevant when categorizing transgenic plants. However, plants regenerated from cultured cells often show a high frequency of alterations in their phenotypes due to somaclonal mutations (Nishi et al. 1968, Heinz and Mee 1969). Thus, information about somaclonal mutations is beneficial in predicting concomitant effects and confirming the elimination of such mutations by backcrossing.

Differences between GM crops and non-GM crops are among the most cited public concerns regarding food safety and security. From this point of view, sequencing and bioinformatics analysis can be used for the molecular characterization of genetically modified crops. For example, the production of an assembled 3× draft genomic sequence (i.e. containing an average depth of sequence coverage of 3×) has been applied to the molecular characterization of a virus-resistant transgenic papaya (*Carica papaya* L.) (Ming et al. 2008). In the case of GM soybean, 75× coverage was adopted to confirm the insertion site of the T-DNA (Kovalic et al, 2012). In addition, transgenic rice expressing a seed-based edible vaccine against Japanese cedar pollinosis was subjected to 11.3–33.2× coverage whole-genome sequencing (Kawakatsu et al. 2013). In the case of GT, T-DNA border sequences, which do not have homology to the plant genome, must be eliminated by HR. Therefore, GT plants are theoretically free from sequences derived from *Agrobacterium*. In this study, we applied Southern blot analysis, PCR analysis, genome CGH array analysis and whole-genome sequencing to the molecular characterization of GT rice plants. This combined analysis detected no sequences derived from *Agrobacterium* in two GT plants, BS^R^-9-9-8 and BS^R^-59-8-5, and somaclonal mutation induced by cell culture within the range of regular mutation breeding. Abiotic transformation methods such as particle gun and polyethylene glycol (PEG) transformation are free from the risk of integration of sequences derived from *Agrobacterium*. However, the disadvantages of these procedures include the damage done to cellular tissue and fragmentation of the transformed DNA. The effects of unpredictable mutations due to DNA damage and studded fragmented exogenous DNA seem to be as risky as incorporation of exogenous sequences. To increase the completeness of whole-genome sequencing, we recommend using a sequencer which can supply long reads with effective increasing read count.

Simultaneous use of custom-designed nucleases such as zinc finger nucleases (ZFNs), transcription activator-like effector nucleases (TALENs) and the clustered regularly interspaced short palindromic repeats (CRISPR)–Cas9 system has been reported to increase GT efficiency drastically in vertebrates (for a review, see Gaj et al. 2013). Enhanced by such techniques, GT is a plant molecular breeding technique that will become widely available in the near future. Furthermore, in the next few years, many regulatory jurisdictions around the world will make decisions on the governance of new plant breeding techniques, such as the site-directed mutagenesis of plant genes (to knock-out or modify gene function) and the targeted deletion or insertion of genes into plant genomes. We hope that our report can be utilized as a test case of the molecular characterization of such new plant breeding products, and that it will contribute to the discussion surrounding the required certification.

## Materials and Methods

### Plant materials

Rice plants containing two point mutations corresponding to W548L and S627I in the rice *ALS* gene generated by HR-dependent GT (Endo et al. 2007) were used in this study. BS^R^-12-1 and BS^R^-12-2 are the T_2_ progeny of the same GT regenerated plant (T_0_). BS^R^-9-9-8 and BS^R^-59-8-5 are the T_3_ progeny of independent GT plants.

### Primers

Primers used in this study are listed in Supplementary Table S5.

### Southern blot analysis

Genomic DNA was extracted from leaves of individual seedlings using the Nucleon Phytopure extraction kit (Amersham Pharmacia Biotech) according to the manufacturer’s instructions. After endonuclease digestion and electrophoresis on a 1% agarose gel, DNA fragments were transferred to a positively charged nylon membrane (Roche). Probes were prepared using a PCR DIG probe synthesis kit (Roche). Hybridization was performed according to the DIG Application Manual (Roche). Hybridization was at 42°C and washing was performed under high-stringency conditions at 68°C.

### CGH array design

We used Agilent’s web portal eArray (https://earray.chem.agilent.com/earray/) to design custom-made arrays with a very high density of probes in the vector sequence. We designed 15K oligonucleotide probes in an 8× 15K format. A total of 15,000 60-mer oligonucleotide probes were designed by delimiting every 60 bp from the single strand of the rice bacterial artificial chromosome (BAC) clone OsJNBa0052M16 and two strands of the binary vector backbone, pPZ2028, overlapping with 5 bp slides. OsJNBa0052M16 is a BAC clone that contains *Oryza sativa* Japonica group chromosome 2 genomic DNA covering the rice genomic sequence located on the T-DNA region of the GT vector. In order to remove systematic biases such as spatial and intensity artifacts, we normalized the Cy5/Cy3 value by dividing the value of each probe by the median value of 13 positive control probes, DarkCorner, SM_01–SM_12 in each array. Cy5-labeled DNAs in arrays No. 1 to No. 6 are as follows: No. 1, WT (Cy5); No. 2, WT with GT vector; No. 3, BS^R^-12-1 (Cy5); No. 4, BS^R^-12-2; No. 5, BS^R^-9-9-8; No. 6, BS^R^-59-8-5. Cy3-labeled DNA was WT in every array.

### Sample labeling and microarray hybridization

All array hybridizations were performed according to the manufacturer’s recommended protocols. Briefly, 1.5 µg of genomic DNA was digested with restriction enzymes *Alu*I and *Rsa*I and labeled fluorescently using the Agilent Genomic DNA Labeling Kit (No. 5188-5309). Test samples were labeled with Cy5, and the reference sample was labeled with Cy3. The labeled DNA fragments were purified using Microcon YM-30 filter units (Millipore). Absorbance was measured at 260 (DNA), 550 (cyanine 3) or 650 nm (cyanine 5) to calculate the specific activity. Array hybridization was performed using the Agilent Oligo CGH/ChIP-on-chip Hybridization Kit (No. 5188-5220) for 40 h at 20 r.p.m. in a 65°C Agilent hybridization oven. Standard wash procedures were then performed. Arrays were scanned at 3 µm resolution using an Agilent scanner, and image analysis was performed using Agilent FEATURE EXTRACTION software, version 10.7 with 90% laser power value and 100% photo multiplier tube.

### Data analysis of genome CGH-array

CGH data sets were analyzed with the DNA Analytics 4.0.76 software package (Agilent Technologies). Data were normalized by the centralization algorithm in DNA Analytics. Generally, CGH data fluorescent ratios were normalized by setting the average log fluorescent ratios to zero. However, this can result in false aberration calls in genomes that have a large number of abnormalities. The fuzzy zero correction was applied to remove putative variant intervals with small average log_2_ ratios. Aberrant regions were determined by the ADM-2 algorithm with a threshold of 4.0. The aberration filter was selected with the following parameters: minimum number of probes in region, 5; minimum absolute average log_2_ ratio for region, 3; maximum aberrations, 10,000; and percentage penetrance per feature, 0. Mean log_2_ ratios represent the following amplifications or deletions: one copy amplification +0.6, two copy amplification ≥+1, one copy deletion −1, two copy deletion ≤−2. Log_2_ values of the Cy5/Cy3 ratio were calculated with moving average of 60, 300, 600 and 1,200 bp.

### Whole-genome sequencing

#### Sample preparation

For the next-generation DNA sequencing of rice, total genomic DNA was prepared from young leaves rice using the CTAB (cetyl trimethylammonium bromide) method (Murray and Thompson 1980). The genomic DNA was fragmented by Covaris LE220. Sequencing libraries were constructed according to the manufacturer’s instructions with an Illumina Genomic DNA Sample Preparation Kit. Short read sequences (76 bases) were generated by Illumina Genome Analyzer IIx with TruSeq SSB Kit v5-GA.

#### Alignment to sequences existing in Agrobacterium

To detect *Agrobacterium* genome sequences, Illumina sequencing data were mapped with BWA (Burrows–Wheeler Aligner, ver.0.6.1-r104) against *Agrobacterium* genome and binary vector sequence data as reference sequences (accession IDs are DQ058764.1, NC_003062.2, NC_003063.2, NC_003064.2 and U10461.1) with default options (Li and Durbin 2009). After sequencing, the Illumina adaptor sequences and low quality data were removed by cutadapt from Illumina fastq data. Then, short sequence data (<14 bases) were eliminated. Illumina sequence data (>15 bases) were used as a query for BWA analyses. Mapping results were evaluated by alignment patterns.

#### Screening of variant candidates

Short-read sequences were mapped to *O. sativa* (japonica cultivar Nipponbare) reference genome IRGSP1.0 (Sakai et al. 2013) by BWA v0.6.1 (Li and Durbin 2009) with the default settings, and only uniquely mapped reads with ≥20 mapping quality were retained. After conducting local re-alignment around the InDels using GATK v1.5 (McKenna et al. 2010), putative PCR duplicate reads were removed by Picard (http://broadinstitute.github.io/picard/). Raw SNPs and short InDels were detected using SAMtools v0.1.18 (Li et al. 2009). Variants were filtered with several conditions by a program, vcfutils.pl, implemented in the SAMtools package. We estimated the effects of variants using snpEff v3.3 (Cingolani et al. 2012) with a database built from the latest version of representative genes of RAP-DB.

## Supplementary data

Supplementary data are available at PCP online.

## Funding

This work was supported by the National Institute of Agrobiological Sciences strategic research fund; a grant from the Development of Genome Information Database System for Innovation of Crop and Livestock Production.

## Supplementary Material

Supplementary Data

## Abbreviations

ALS: acetolactate synthase
ASA2: anthranilate synthase α-subunit 2
BAC: bacterial artificial chromosome
BS: bispyribac
CAPS: cleaved amplified polymorphic sequence
CTAB: cetyl trimethylammonium bromide
Cy5: cyanine 5-dUTP
Cy3: cyanine 3-dUTP
CGH: comparative genomic hybridization
GM: genetically modified
GT: gene targeting
HR: homologous recombination
InDel: insertion/deletion
LB: left border
NHEJ: non-homologous end-joining
NGS: next-generation sequencing
RB: right border
SNP: single nucleotide polymorphism
WT: wild-type
