# Supplementary material for: Whole-Genome Analysis of Herbicide-Tolerant Mutant Rice Generated by Agrobacterium-Mediated Gene Targeting
Source: Plant Cell Physiol. 2014 Nov 4;56(1):116–25. doi: 10.1093/pcp/pcu153 (PMC4301741; doi:10.1093/pcp/pcu153)
Supplement: Supplementary Data [file supp_56_1_116__index.html]

Whole genome analysis of herbicide-tolerant mutant rice generated by Agrobacterium-mediated gene targeting — Whole-Genome Analysis of Herbicide-Tolerant Mutant Rice Generated by Agrobacterium-Mediated Gene Targeting — Whole-Genome Analysis of Herbicide-Tolerant Mutant Rice Generated by Agrobacterium-Mediated Gene Targeting — Supplementary Data 

# Whole-Genome Analysis of Herbicide-Tolerant Mutant Rice Generated by *Agrobacterium*-Mediated Gene Targeting

## Supplementary Data

files

**Files in this Data Supplement:**

- Supplementary Data - pdf file
