# Supplementary material for: Whole-Genome Analysis of Herbicide-Tolerant Mutant Rice Generated by Agrobacterium-Mediated Gene Targeting
Source: Plant Cell Physiol. 2014 Nov 4;56(1):116–25. doi: 10.1093/pcp/pcu153 (PMC4301741; doi:10.1093/pcp/pcu153)
Supplement: Supplementary Data [file supp_pcu153_pcp-2014-e-00527-File003.pdf]

Supplementary Table S1. Detection of sequences derived from *Agrobacterium*.

| | | | | trimmed | | all | | % | | mapped (100% match) | | | | mapped (uniq loci) | | | | mapped (alli) | | | |
|---|---|---|---|---|---|---|---|---|---|---|---|---|---|---|---|---|---|---|---|---|---|
| Sample | Reference accession No. | Reference description | Reference length (bp) | Read count | Read length | Read count | Read length | Read count | Read length | Mapped reads | % | Total read length | % | Mapped reads | % | Total read length | % | Mapped reads | % | Total read length | % |
| | DQ058764.1 | Ti plasmid pTiBo542 | 244978 | | | | | | | 0 | 0 | 0 | 0 | 0 | 0 | 0 | 0 | 0 | 0 | 0 | 0 |
| | NC_003062.2 | C58 chromosome circular | 2841580 | | | | | | | 0 | 0 | 0 | 0 | 10 | 2E-05 | 169 | 5E-06 | 254 | 5E-04 | 18713 | 5E-04 |
| $BS^R$-12-1 (T2) | NC_003063.2 | C58 chromosome linear | 2075577 | 47857496 | 3.634E+09 | 48091545 | 3.655E+09 | 99.51 | 99.42 | 0 | 0 | 0 | 0 | 6 | 1E-05 | 103 | 3E-06 | 252 | 5E-04 | 18679 | 5E-04 |
| | NC_003064.2 | C58 plasmid At. | 542868 | | | | | | | 0 | 0 | 0 | 0 | 0 | 0 | 0 | 0 | 0 | 0 | 0 | 0 |
| | U10461.1 | binary vector (pPZP202) | 7132 | | | | | | | 1146 | 0.002 | 86966 | 0.002 | 1469 | 0.003 | 111493 | 0.003 | 1469 | 0.003 | 111493 | 0.003 |
| | DQ058764.1 | Ti plasmid pTiBo542 | 244978 | | | | | | | 0 | 0 | 0 | 0 | 0 | 0 | 0 | 0 | 0 | 0 | 0 | 0 |
| | NC_003062.2 | C58 chromosome circular | 2841580 | | | | | | | 0 | 0 | 0 | 0 | 1 | 4E-06 | 17 | 8E-07 | 112 | 4E-04 | 8311 | 4E-04 |
| $BS^R$-12-2 (T2) | NC_003063.2 | C58 chromosome linear | 2075577 | 26777904 | 2.033E+09 | 26872457 | 2.042E+09 | 99.65 | 95.56 | 0 | 0 | 0 | 0 | 3 | 1E-05 | 47 | 2E-06 | 112 | 4E-04 | 8311 | 4E-04 |
| | NC_003064.2 | C58 plasmid At. | 542868 | | | | | | | 0 | 0 | 0 | 0 | 0 | 0 | 0 | 0 | 0 | 0 | 0 | 0 |
| | U10461.1 | binary vector (pPZP202) | 7132 | | | | | | | 364 | 0.001 | 27638 | 0.001 | 433 | 0.002 | 32882 | 0.002 | 433 | 0.002 | 32882 | 0.002 |
| | DQ058764.1 | Ti plasmid pTiBo542 | 244978 | | | | | | | 0 | 0 | 0 | 0 | 2 | 4E-06 | 32 | 8E-07 | 2 | 4E-06 | 32 | 8E-07 |
| | NC_003062.2 | C58 chromosome circular | 2841580 | | | | | | | 0 | 0 | 0 | 0 | 1 | 2E-06 | 16 | 4E-07 | 524 | 9E-04 | 39628 | 9E-04 |
| $BS^R$-9-9-8 (T3) | NC_003063.2 | C58 chromosome linear | 2075577 | 55633580 | 4.224E+09 | 56205201 | 4.272E+09 | 98.98 | 98.89 | 0 | 0 | 0 | 0 | 2 | 4E-06 | 31 | 7E-07 | 524 | 9E-04 | 39628 | 9E-04 |
| | NC_003064.2 | C58 plasmid At. | 542868 | | | | | | | 0 | 0 | 0 | 0 | 0 | 0 | 0 | 0 | 1 | 2E-06 | 16 | 4E-07 |
| | U10461.1 | binary vector (pPZP202) | 7132 | | | | | | | 0 | 0 | 0 | 0 | 0 | 0 | 0 | 0 | 0 | 0 | 0 | 0 |
| | DQ058764.1 | Ti plasmid pTiBo542 | 244978 | | | | | | | 0 | 0 | 0 | 0 | 0 | 0 | 0 | 0 | 0 | 0 | 0 | 0 |
| | NC_003062.2 | C58 chromosome circular | 2841580 | | | | | | | 0 | 0 | 0 | 0 | 1 | 2E-06 | 76 | 2E-06 | 311 | 7E-04 | 23596 | 7E-04 |
| $BS^R$-59-8-5 (T3) | NC_003063.2 | C58 chromosome linear | 2075577 | 43011428 | 3.266E+09 | 43374403 | 3.296E+09 | 99.16 | 99.07 | 0 | 0 | 0 | 0 | 1 | 2E-06 | 17 | 5E-07 | 311 | 7E-04 | 23537 | 7E-04 |
| | NC_003064.2 | C58 plasmid At. | 542868 | | | | | | | 0 | 0 | 0 | 0 | 1 | 2E-06 | 16 | 5E-07 | 1 | 2E-06 | 16 | 5E-07 |
| | U10461.1 | binary vector (pPZP202) | 7132 | | | | | | | 0 | 0 | 0 | 0 | 0 | 0 | 0 | 0 | 0 | 0 | 0 | 0 |

Suppmenentary Table S2. Number of mutation detected by whole genome sequencing*.

| Sample (generation) | hom SNP | het SNP | hom InDel | het InDel | Total |
|---|---|---|---|---|---|
| $BS^R$-12-1 (T2) | 5,446 | 11,309 | 1,220 | 928 | 18,903 |
| $BS^R$-12-2 (T2) | 5,915 | 11,464 | 810 | 490 | 18,679 |
| $BS^R$-9-9-8 (T3) | 4,693 | 8,627 | 1,154 | 675 | 15,149 |
| $BS^R$-59-8-5 (T3) | 4,766 | 8,100 | 868 | 479 | 14,213 |
| Shared variant** | 10,757 | | 1,556 | | 12,313 |

* Raw SNPs and InDels detection by SAMtools.

** Shared by at least 2 sequenced sample except the combination of $BS^R$-12-1 and $BS^R$-12-2.

Supplementary Table S3. Number of unshared variants*

| ** | homSNP | hetSNP | homINDEL | hetINDEL | Total |
|---|---|---|---|---|---|
| Qual >= 10 | | | | | |
| $BS^R$-12-1 (T2) | 355 | 1533 | 258 | 410 | 2556 |
| $BS^R$-12-2 (T2) | 270 | 838 | 125 | 208 | 1441 |
| $BS^R$-9-9-8 (T3) | 300 | 882 | 180 | 226 | 1588 |
| $BS^R$-59-8-5 (T3) | 239 | 515 | 93 | 142 | 989 |
| Qual >= 20 | | | | | |
| $BS^R$-12-1 (T2) | 253 | 830 | 223 | 220 | 1526 |
| $BS^R$-12-2 (T2) | 187 | 443 | 109 | 94 | 833 |
| $BS^R$-9-9-8 (T3) | 213 | 522 | 152 | 120 | 1007 |
| $BS^R$-59-8-5 (T3) | 169 | 264 | 72 | 51 | 556 |
| Qual >= 30 | | | | | |
| $BS^R$-12-1 (T2) | 185 | 466 | 139 | 132 | 922 |
| $BS^R$-12-2 (T2) | 146 | 198 | 57 | 37 | 438 |
| $BS^R$-9-9-8 (T3) | 172 | 314 | 100 | 61 | 647 |
| $BS^R$-59-8-5 (T3) | 129 | 125 | 42 | 18 | 314 |

*Variant remaining after excluding PCR duplicates, filtering raw quality variants, subtracting variants shared by several samples.
**Different quality call( >=10, 20, 30) with DP>=2 at SAMtools were applied to selecting unshared variants.

Supplementary Table S4. Coverage of the genome*.

| chr | chr length | Depth >=2, Quality 10 covered site | coverage (%) | Depth >=4, Quality 20 covered site | coverage (%) |
|---|---|---|---|---|---|
| chr01 | 43,270,923 | 31592180 | 73 | 20891958 | 48.3 |
| chr02 | 35,937,250 | 26772323 | 74.5 | 17683979 | 49.2 |
| chr03 | 36,413,819 | 27418075 | 75.3 | 18156968 | 49.9 |
| chr04 | 35,502,694 | 23709550 | 66.8 | 15099454 | 42.5 |
| chr05 | 29,958,434 | 20771066 | 69.3 | 13512475 | 45.1 |
| chr06 | 31,248,787 | 21919358 | 70.1 | 14254422 | 45.6 |
| chr07 | 29,697,621 | 20892498 | 70.4 | 13563821 | 45.7 |
| chr08 | 28,443,022 | 19763867 | 69.5 | 12785952 | 45 |
| chr09 | 23,012,720 | 16215634 | 70.5 | 10475382 | 45.5 |
| chr10 | 23,207,287 | 16040477 | 69.1 | 10292352 | 44.3 |
| chr11 | 29,021,106 | 20176745 | 69.5 | 12957765 | 44.6 |
| chr12 | 27,531,856 | 18824780 | 68.4 | 12050065 | 43.8 |
| **Total** | **373,245,519** | **264096553** | **70.8** | **171724593** | **46** |

* Coverage of the genome shared by 4 sequenced samples

Supplementary Table S5. Primer list

| Name | Sequence (5'-3') |
|---|---|
| F1 | CGTCACCGCGCGCGGACAAAACACCCAC |
| R1 | ACATGATATCTTGTGATGCATATGCCTAC |
| pPZP1 | CTTTCTCATAGCTCACGCTGTAGGTATCTCA |
| pPZP2 | GATGCACTAAGCACATAATTGCTCACAG |
| pPZP3 | AGGGCGTGAAAAGGTTTATCCGTTCGTCCA |
| pPZP4 | AACGTCGCCAGGGCGTAGGTGGTCAAGCAT |

Sup Fig. S1

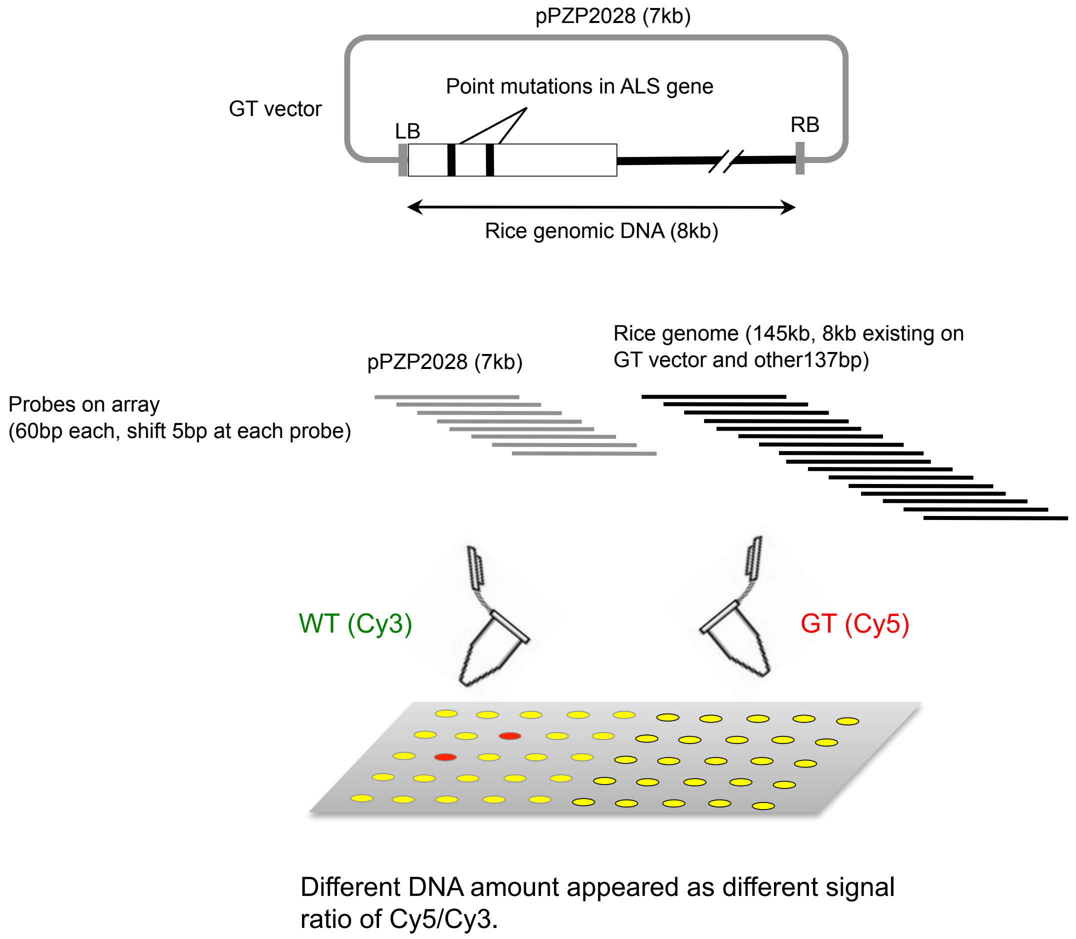


**Supplymentary Figure S1** Design of custom-made CGH array

A total of 15,000 60-mer oligo-nucleotide probes were designed by delimiting each 60 bp from a single strand of the rice BAC clone OsJNBa0052M16 and double strands of the binary vector pPZ2028 (7kb). OsJNBa0052M16 contains 145kb of *Oryza sativa* Japonica group chromosome 2 genomic DNA and rice genome located on the T-DNA region of the GT vector that exists on this BAC clone. All the sequences were divided into 60 bp fragments overlapping with 5bp slides. Out of 60bp, 55bp probes are overlapping with next probe. Equal amounts of genomic DNA from WT rice plants and GT plants were labeled with either cyanine 5-dUTP (Cy5) or cyanine 3-dUTP (Cy3), and hybridized to the custom-made array. In each genome CGH-array experiment, genomic DNA from WT was labeled by Cy3. DNA samples labeled by Cy5 in each array experiment were as follows: Array Nos. 1, WT; No.2, WT rice genome mixed with GT vector; No.3, $BS^R$-12-1; No.4, $BS^R$-12-2. No.5, $BS^R$-9-9-8; No.6, $BS^R$-59-8-5.

# Sup Fig. S2

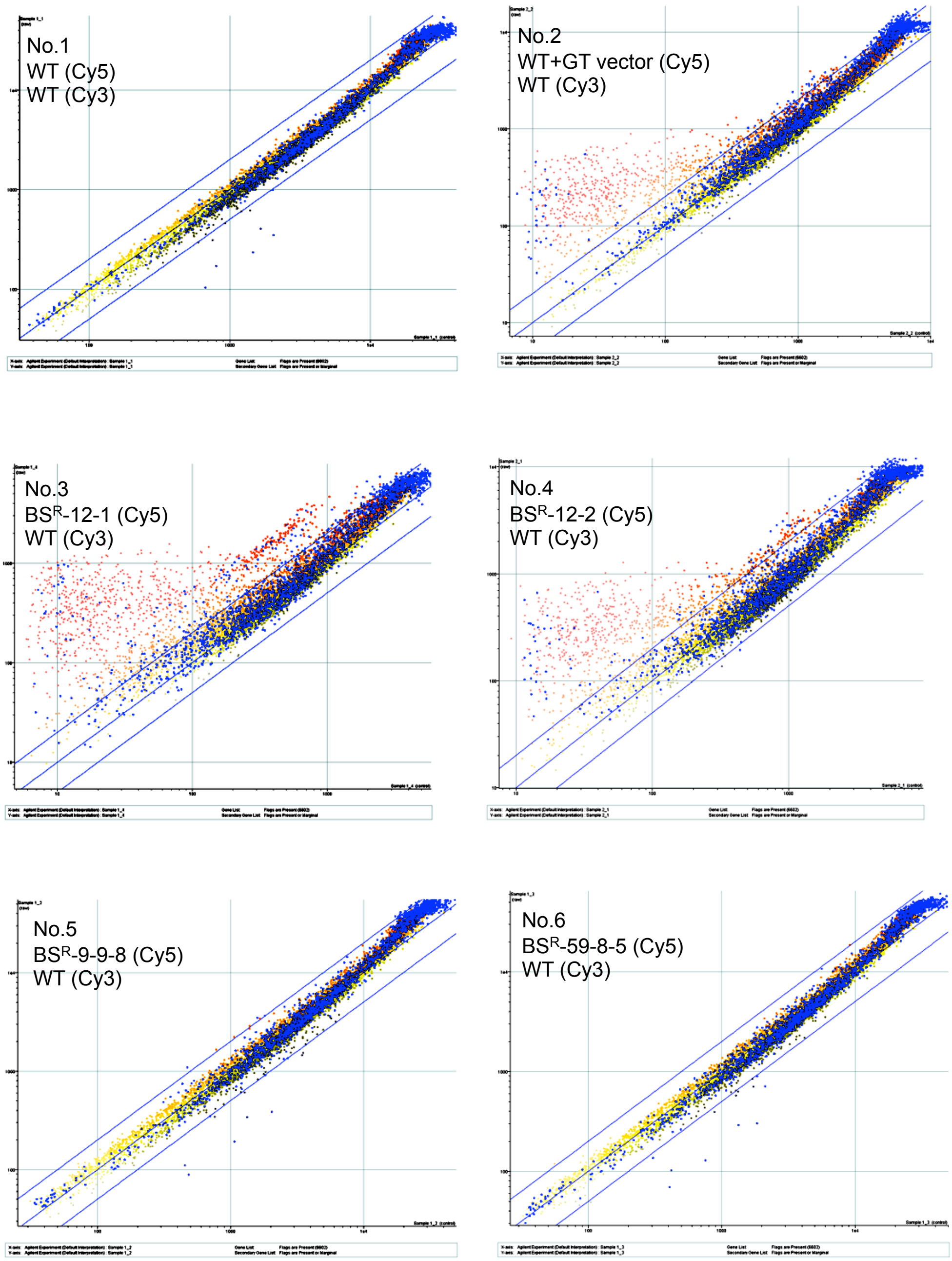


**Suppmenentary Figure S2** Scatter plot of genome CGH-array results

Comparison of signal intensities of each probe between Cy5 labeled DNA (*Y*-axis) and Cy3 labeled DNA (*X*-axis) . The three blue diagonal lines indicate ratios of the sample to the reference intensities of 2, 1, and 1/2 from left to right, respectively.

Sup Fig. S3

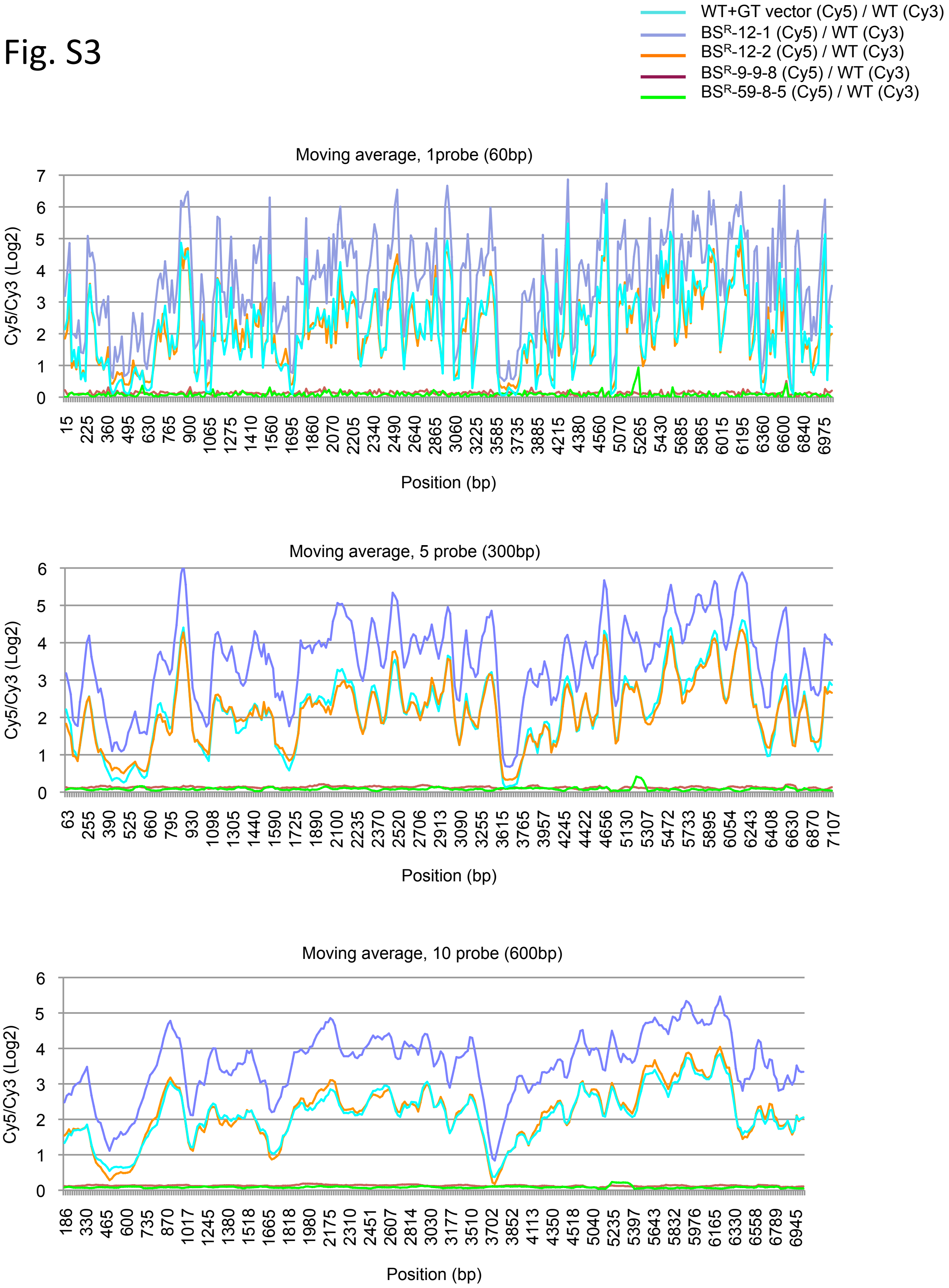


**Supplementary Figure S3** Array CGH ratio plots for the forward strand of binary vector, pPZP2028

The log2 ratios of Cy5/Cy3 using a moving average of 1, 5, 10 adjacent probes were plotted against the cumulative kilobase pairs positioning.

# Sup Fig. S4

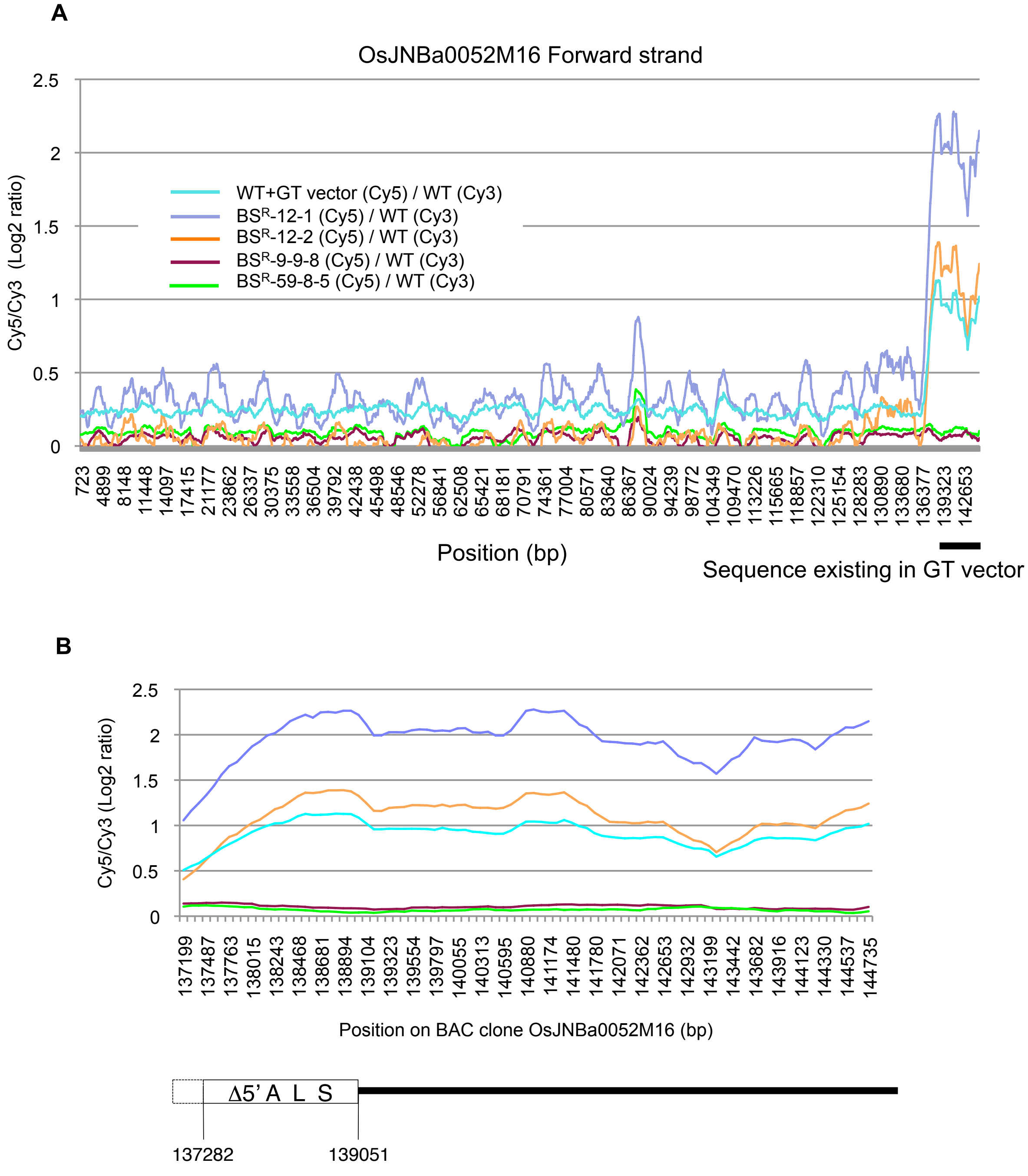


**Suppmenentary Figure S4** Array CGH ratio plots for the rice genome

(A) The log2 ratios of Cy5/Cy3 using a moving average of 20 adjacent probes (1.2kb) were plotted against the cumulative kilobase pairs positioning of rice BAC clone OsJNBa0052M16. Location of the sequence existing in the gene vector is shown below the graph. ALS ATG, 137116 bp; GT vector start, 137282 bp; ALS stop, 139051 bp; GT vector end, 145373 bp (B) Magnified view of the rice genomic region on GT vector.

# Sup Fig. S5

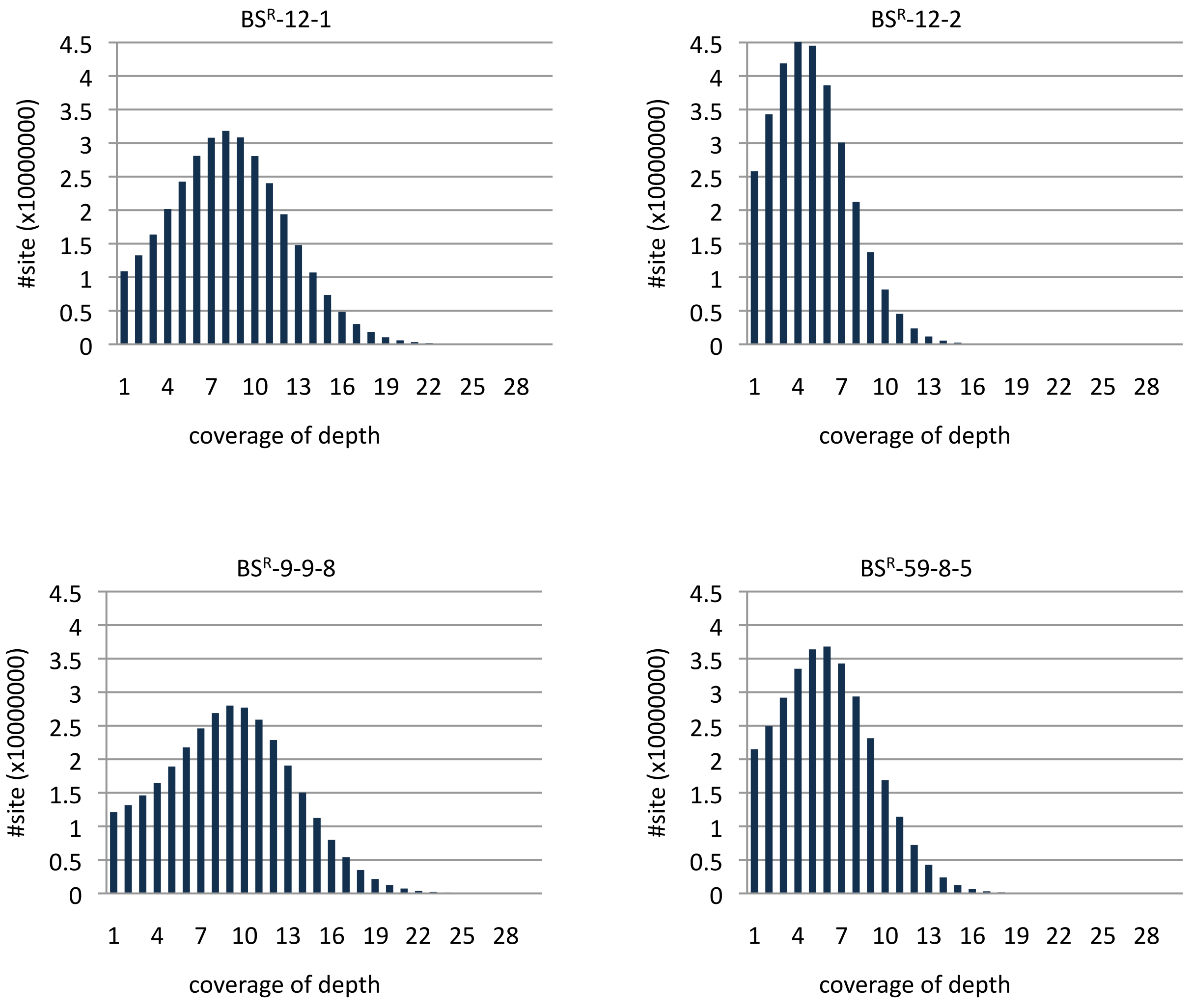


**Supplementary Figure S5** Distribution chart of depth of sequencing

Number of reads observed for each read depth is shown. The peaks of sequencing depth of BS$^R$-12-1, BS$^R$-12-2, BS$^R$-9-9-8 and BS$^R$-59-8-5 were 8, 4, 10, and 6, respectively.
